# An Evaluation of the Effectivity of the Green Leaves Biostimulant on Lettuce Growth, Nutritional Quality, and Mineral Element Efficiencies under Optimal Growth Conditions

**DOI:** 10.3390/plants13070917

**Published:** 2024-03-22

**Authors:** Santiago Atero-Calvo, María José Izquierdo-Ramos, Carmen García-Huertas, Miguel Rodríguez-Alcántara, Iván Navarro-Morillo, Eloy Navarro-León

**Affiliations:** 1 Department of Plant Physiology, Faculty of Sciences, University of Granada, 18071 Granada, Spain; satero@ugr.es (S.A.-C.); mariajoseir@ugr.es (M.J.I.-R.); cgarciahuertas@correo.ugr.es (C.G.-H.); miguel1632002@correo.ugr.es (M.R.-A.); 2 R + D Department of Atlántica Agrícola, 03400 Villena, Spain; inavarro@atlanticaagricola.com

**Keywords:** antioxidant compounds, biostimulant, glycine betaine, lettuce, nutrient use efficiency, photosynthesis

## Abstract

The use of biostimulants is becoming a useful tool for increasing crop productivity while enhancing nutritional quality. However, new studies are necessary to confirm that the joint application of different types of biostimulants, together with bioactive compounds, is effective and not harmful to plants. This study examined the impact of applying the biostimulant Green Leaves, comprising *Macrocystis* algae extract and containing a mixture of amino acids, corn steep liquor extract, calcium, and the bioactive compound glycine betaine. The effect of applying two different doses (3 and 5 mL L^−1^) of this biostimulant was evaluated on lettuce plants, and growth and quality parameters were analyzed along with photosynthetic efficiency, nutritional status, and nutrient efficiency parameters. The application of Green Leaves improved plant weight (25%) and leaf area and enhanced the photosynthetic rate, the accumulation of soluble sugars and proteins, and the agronomic efficiency of all essential nutrients. The 3 mL L^−1^ dose improved the nutritional quality of lettuce plants, improving the concentration of phenolic compounds and ascorbate and the antioxidant capacity and reducing NO_3_^−^ accumulation. The 5 mL L^−1^ dose improved the absorption of most nutrients, especially N, which reduced the need for fertilizers, thus reducing costs and environmental impact. In short, the Green Leaves product has been identified as a useful product for obtaining higher yield and better quality.

## 1. Introduction

In contemporary agriculture, the use of products to enhance crop development and mitigate the effects of environmental stresses has become widespread. Consequently, biostimulants are increasingly employed to influence the physiological processes of plants, thereby optimizing their performance [1,2,3]. The characteristics of biostimulants, their bioactive components, the explanation of their action mechanisms, and their effects on plants across morphological, biochemical, and metabolic dimensions are the foci of many scientists, industrial companies, and farmers [4]. Plant biostimulants are defined as any product that boosts plant development and nutritional status, without dependence on their nutrient content, to improve the efficient use of nutrients, tolerance to abiotic stress, and qualitative characteristics [5,6,7]. Biostimulants contribute to enhanced plant growth by increasing photosynthesis, germination, the absorption and utilization of nutrients in plants, and the concentration of growth hormones, as well as reducing senescence, which increases plant quality, productivity, and post-harvest useful life [3,8,9].

Within the diverse range of biostimulants available, those derived from amino acids (AAs) and algae extracts are particularly prevalent. The beneficial impact of amino acid application on plant growth is well documented, as these compounds contribute to the synthesis of numerous non-protein nitrogenous substances (such as pigments, vitamins, coenzymes, and nitrogen bases) [10]. Likewise, seaweed is recognized for its valuable contribution to enhancing the growth of a wide array of crops, offering both direct and indirect advantages [9]. The interest in seaweed extracts as biostimulants has surged due to their rich biochemical composition, which includes a broad spectrum of bioactive elements and signaling molecules like phytohormones, vitamins, Aas, polysaccharides, and micro and macronutrients [11,12]. These types of biostimulants have positive effects on plants by modifying the root architecture, improving the efficiency of nutrient use, especially nitrogen (N), and inducing the assimilation of nitrates, therefore reducing their accumulation in the leaves. Furthermore, they can promote resistance to unfavorable environmental conditions by increasing tolerance to stresses through the induction of antioxidant and osmoprotective compounds [10,13,14,15]. Various studies have verified the biostimulant effect in plants after individual application of extracts based on AAs and algae, although research into the effect of the joint application of these biostimulants is scarcer [9,16].

In addition, a series of bioactive materials or bioregulators are usually added to biostimulant formulations to improve the beneficial effects of these products. Among the most used compounds are phytohormones, antioxidant compounds such as ascorbate and α-tocopherol, nitrogenous compounds such as proline, glycine betaine, and polyamines, sugars and polyalcohols such as trehalose and mannitol, and macronutrients such as calcium (Ca) [17]. However, most of the beneficial effects have primarily been noted in plants experiencing stress, while plants cultivated under control conditions showed less pronounced benefits, and in some cases, even adverse effects were reported, including diminished growth. For instance, in *Arabidopsis thaliana*, and in several horticultural crops, the application of compounds such as proline, ascorbate, melatonin, and tocopherol under standard growth conditions resulted in decreased plant growth [18].

Currently, many studies on biostimulants are being conducted on lettuce [19,20,21], as it is a key leafy vegetable in global diets and is rich in vitamins, minerals, and dietary fiber, which significantly contribute to human nutrition and health [22]. Numerous studies have demonstrated that the application of plant biostimulants to lettuce effectively enhances fresh weight, leaf area, and, consequently, yields [19,20,23,24]. Furthermore, biostimulants significantly improve lettuce quality by enhancing color and antioxidant content [21,23]. Additionally, they facilitate the efficient utilization of nutrients, allowing for the reduction of fertilizers and promoting the uptake of macronutrients such as N, Ca, magnesium (Mg), and phosphorus (P), as well as micronutrients like iron (Fe), thereby augmenting nutritional quality [19,24,25].

In summary, conducting research is essential to verify that the combined use of biostimulants and bioactive compounds, while ensuring no phytotoxic effects on plants under suitable conditions, has a positive impact on their growth and development. However, there is no clear information. Moreover, there is still a lack of definitive information regarding the impact of these types of biostimulants on plant physiology and nutritional efficiencies. Unlike previous research focused on biostimulants based on a single type of extract or compound, this study explores the synergistic effects of a new product called Green Leaves, a biostimulant comprising a blend of *Macrocystis* alga extract, amino acids, steeped corn liquor extract, calcium, and the bioactive compound glycine betaine. Therefore, the main objective of this study is to analyze the effect of Green Leaves on *Lactuca sativa* L. cv. Maravilla de Verano plants grown under adequate growth conditions. The effect of applying different doses of this biostimulant on lettuce plants was evaluated, and growth and quality parameters were analyzed along with the study of basic physiological processes of primary metabolism, such as photosynthetic efficiency, nutritional status, and nutrient efficiency parameters.

## 2. Results and Discussion

### 2.1. Growth Parameters

A fundamental attribute of a biostimulant, as highlighted by [26], is its non-toxicity to plants alongside its capacity to enhance plant growth and yield. This research evaluates factors such as the generation of fresh and dry biomass in the plant’s aerial sections and leaf area, which were analyzed to assess the effect of the product Green Leaves on biomass production. These metrics serve as reliable indicators of plant development across various cultivation environments [1,16].

**Figure 1:**
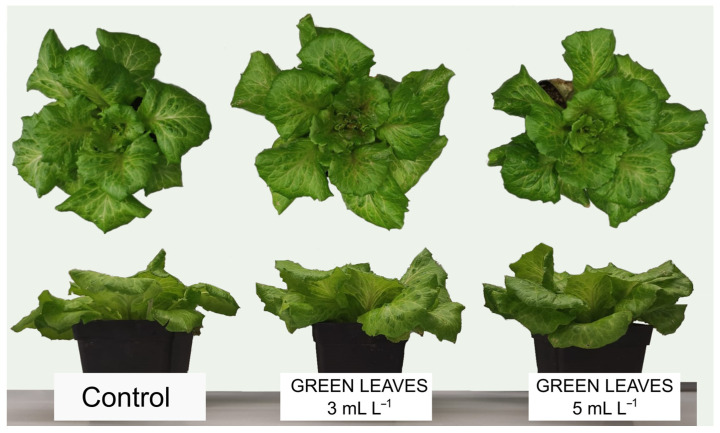
Front and top views of lettuce plants subjected to different treatments at sampling time.

Foliar application of Green Leaves at two doses (3 and 5 mL L^−1^) produced positive effects by increasing biomass production compared with the control plants, with no significant differences between both treatments (Table 1 and Figure 1). Regarding the percentage of increase in the production of fresh biomass of the shoot, the application of Green Leaves increased growth compared with the control plants by 23% for the 3 mL L^−1^ dose and by 26% for the 5 mL L^−1^ dose (Table 1); therefore, both doses presented similar effects. However, considering dry weight, there were greater differences in percentage between the doses applied, with the dose of 3 mL L^−1^ being the one that gave rise to the most biostimulant effect, with an increase of 46% compared with control plants, whereas the application of 5 mL L^−1^ produced a lower increase of 36% (Table 1). Regarding the values of leaf area values, all treatments with the Green Leaves product significantly improved this parameter, with no statistically significant differences between the two doses applied (Table 1, Figure 1). Therefore, the application of Green Leaves, especially at a dose of 3 mL L^−1^, could produce a significant increase in the photosynthetic performance of plants by increasing the available area for light capture.

Other experiments that used biostimulants derived from *Macrocystis* algae extracts also found biomass increments in lettuce plants [27,28]. Furthermore, the addition of other components to the Green Leaves product, such as corn steep liquor, macronutrients such as Ca, and AAs such as glutamic acid and glycine, have been proven to have a positive effect on plant growth [19,24,29,30]. However, regarding glycine betaine, it has been found that its positive effects are predominantly observed in plants under stress conditions, while in those grown in optimal environments, the benefits are less noticeable, with some instances of adverse effects reported [18]. Our results confirm that the use of glycine betaine in the Green Leaves product, when used in suitable growing conditions without any stress factors, does not affect the biostimulant effect of this product at any of the doses used. Therefore, its use is highly recommended.

### 2.2. Photosynthesis-Related Parameters

To connect plant growth enhancement and the triggering or boosting of various fundamental physiological processes within plants, this research focuses on examining photosynthesis performance through the analysis of photochemical activity through chlorophyll a (Chl *a*) fluorescence analysis and the efficiency of photosynthesis through gas exchange parameters [31,32]. It was proved that Chl *a* fluorescence is indicative of the plant’s photochemical status and the photosynthetic alterations caused by stress. Therefore, any metabolic disruption leads to the emission of fluorescence by the plant as a means to disperse surplus energy and mitigate stress-induced harm [32]. A key measure obtained from Chl *a* fluorescence analysis is the quantum yield of primary photosynthesis (Fv/Fm), which reliably reflects the photosynthetic efficiency of a plant. Typically, in plants that are not exposed to severe stress, the Fv/Fm ratio remains around 0.85, reflecting healthy photosynthetic activity [33]. In the present experiment, the Fv/Fm index for the treatments with Green Leaves at all doses presented values around 0.87, similar to those of the control plants, which indicates that the application of this product at the applied doses did not cause photoinhibition (Table 2). Other indices of photochemical performance indicate different aspects of photochemical activity, such as the proportion of active reaction centers (RC/ABS), the efficiency of electron transport (Ψ_o_), and the global performance index (PI_ABS_) [32]. In our study, no differences were observed between the differently applied treatments for these parameters, indicating no effects on photochemical activity by Green Leaves application (Table 2).

The concentrations of Chl *a*, *b*, and carotenoids, along with the information derived from Chl *a* fluorescence analysis, reflect the plant photosynthetic activity and photochemical performance [34]. The application of Green Leaves at both doses (3 and 5 mL L^−1^) did not produce statistically significant changes in the foliar concentration of photosynthetic pigments, presenting values similar to those of control plants (Table 3). Ultimately, these data suggest that Green Leaves does not enhance the process of light absorption nor the conversion of light energy into chemical energy, indicating a lack of beneficial impact in these areas.

In the analysis of gas exchange parameters, other studies have shown that if the application of algae-derived biostimulants is effective, it leads to an increase in net photosynthetic rate (*A*). However, the effect on transpiration is more variable, with varying results depending on the experimental conditions [20,31,35]. In the present experiment, both applied doses of Green Leaves increased *A* and intercellular CO_2_ (C_i_) compared with the minimum values presented in control plants (Table 4). Regarding the parameters related to transpiration, we observed that Green Leaves applied at 3 and 5 mL L^−1^ doses produced a decrease in stomatal resistance (r) (Table 4), therefore favoring stomatal opening, which could explain the higher transpiration rate (*E*) compared to control plants. However, this greater transpiration produced by the application of Green Leaves did not represent a significant decrease in water use efficiency (WUE), because these plants presented values similar to those obtained in control plants (Table 4). Although more research must be conducted, the results of photosynthetic efficiency suggest that the application of Green Leaves could be a useful strategy in conditions of water and/or saline stress to improve the adaptation of plants to these stress conditions, especially in leaf crops that are very sensitive to these conditions because the use of Green Leaves improved *A* while maintaining constant WUE values (Table 4).

**Figure 2:**
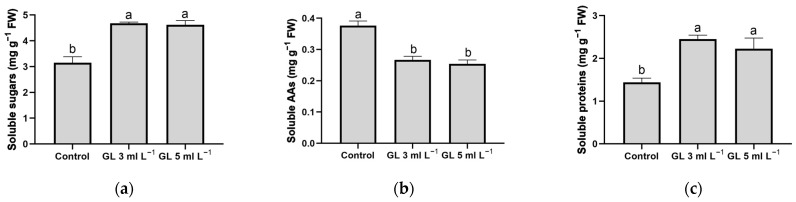
Effect of Green Leaves treatments on the concentration of soluble sugars (**a**), soluble amino acids (AAs) (**b**), and soluble proteins (**c**) in lettuce leaves. Columns represent the means, and error bars represent standard error. Values with different letters indicate significant differences.

The enhancement of photosynthesis in plants activates carbon metabolism and produces carbohydrates vital for plant development. These substances are crucial because they supply the energy and carbon frameworks necessary for the creation of biomolecules for plant growth [36]. In general, the application of both doses of Green Leaves produced a significant increase in the concentration of soluble sugars in the plants compared with control plants (Figure 2a), which is related to the higher photosynthetic activity (Table 4) and the enhanced production of biomass and leaf area (Table 1). Other studies in rice and tomato plants also suggest that biostimulant application enhanced photosynthesis performance, which in turn increased soluble sugar accumulation [37,38].

### 2.3. Soluble Amino Acids, and Protein Concentrations

Analyzing soluble AAs is crucial due to their pivotal roles in primary metabolism. These roles include the synthesis of Chl, growth hormones, and proteins, as well as the regulation of water relations and the sustenance of photosynthetic processes. Furthermore, some of the essential AAs intervene in the induction of secondary metabolism, generating antioxidant and defense compounds such as phenols, alkaloids, etc. [39,40,41]. Foliar application of the Green Leaves product at both doses (3 and 5 mL L^−1^) reduced the foliar concentration of soluble AAs compared with the maximum values obtained in control plants (Figure 2b). This result is not negative because an increase in the accumulation of soluble AAs indicates increased proteolysis and stress in plants [42,43]. Although one of the basic components of the Green Leaves product is a mixture of AAs, it is likely that these AAs applied exogenously in the Green Leaves product are being used in primary metabolism functions, which would explain the greater growth of lettuce plants (Table 1) and the induction, for example, of photosynthetic efficiency (Table 4).

Besides soluble amino acids, evaluating the foliar levels of soluble proteins is crucial for understanding changes in plant growth. Soluble proteins indicate the presence of cellular enzymes critical for fundamental activities like nitrogen assimilation, photosynthesis, and carbon metabolism [39,40]. In the present experiment, the application of both doses of Green Leaves resulted in a significant increase in the concentration of soluble proteins (Figure 2c), suggesting that the externally applied AAs in this product could be utilized efficiently by the plants. This utilization likely accounts for the observed growth enhancement in plants treated with Green Leaves (Table 1). The findings from other research align with our observations, demonstrating that biostimulant applications in rice and bean crops lead to an increase in soluble proteins and a decrease in certain AAs, such as proline [4,38].

### 2.4. Antioxidant Compounds and Capacity

Numerous compounds, such as phenolics and ascorbate, have antioxidant activity that, in addition to increasing tolerance to stress, enhance the nutritional quality of crops [44]. Various studies have found that the application of biostimulants derived from algae can increase the accumulation of these types of compounds and the global antioxidant capacity [45,46,47]. The results of the present experiment suggest that it was mainly the application of the 3 mL L^−1^ dose of the Green Leaves product that produced a higher foliar concentration of total phenols and anthocyanins, followed by the application of Green Leaves at the 5 mL L^−1^ dose, with an increase in their levels when both doses were applied compared with the control plants. In the case of flavonoids, both doses of the biostimulant increased their concentration equally in lettuce plants (Table 5). The other important antioxidant compound analyzed was ascorbate, also known as vitamin C, which is key for antioxidant protection to detoxify ROS and participate in the ascorbate–glutathione cycle. In addition, humans are incapable of synthesizing it; therefore, their needs depend entirely on its consumption through diet [48]. In this study, similar results to the total phenol concentration were observed for ascorbate concentration, with the plants supplied with the 3 mL L^−1^ Green Leaves dose having higher ascorbate accumulations (Table 5).

**Figure 3:**
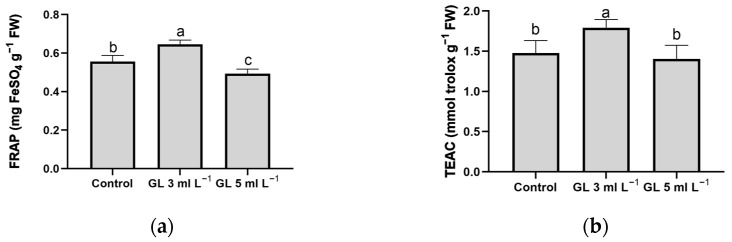
Effect of treatments on antioxidant test values FRAP (**a**) and TEAC (**b**) in leaves of lettuce plants. Columns represent the means, and error bars represent the standard error. Values with different letters indicate significant differences.

To determine the antioxidant capacity as accurately as possible, an analysis of different antioxidant tests was performed [49]. Our results suggest that the 3 mL L^−1^ dose of Green Leaves was the only effective treatment to induce a significant increase in the antioxidant capacity of lettuce plants (Figure 3). The application of the 5 mL L^−1^ dose, although it produced increases in the concentrations of the antioxidant compounds previously described (Table 5), did not lead to a significant increase in the antioxidant capacity defined through the Ferric Reducing Antioxidant Power (FRAP) and Trolox Equivalent Antioxidant Activity (TEAC) test compared with the control plants (Figure 3). In the plants supplied with the 5 mL L^−1^ dose, the observed increases in antioxidant compounds (Table 5) could not be high enough to increase the antioxidant capacity of these plants. Additionally, a saturation of response mechanisms or an interaction with other elements of the metabolic pathway for the biosynthesis of these compounds could occur [50].

### 2.5. NO_3_^−^ Concentration

**Figure 4:**
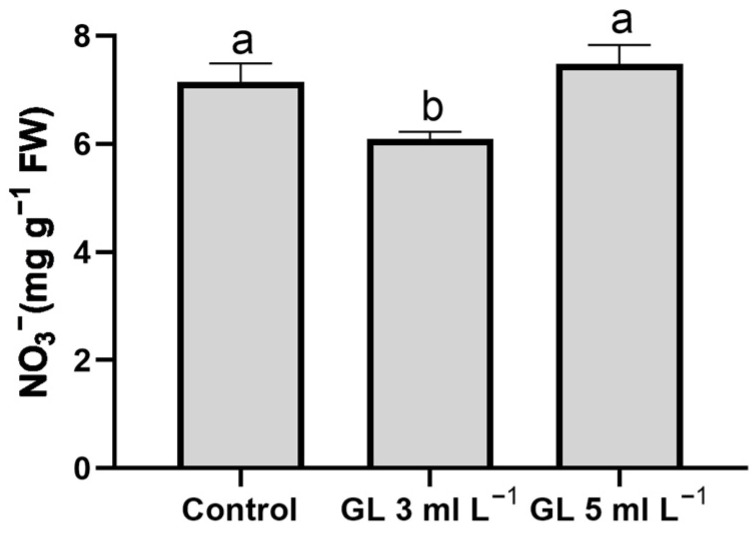
Effect of treatments on nitrate concentration in the leaves of lettuce plants. Columns represent the means, and error bars represent the standard error. Values with different letters indicate significant differences.

In leafy vegetables such as lettuce, understanding leaf NO_3_^−^ levels is crucial because of their potential health hazards. Once ingested, NO_3_^−^ can rapidly convert into nitrite and N-nitroso compounds, which are harmful. These substances can lead to serious health issues, including methemoglobinemia and an elevated risk of cancer [51]. Our results indicate that Green Leaves application, especially at the 3 mL L^−1^ dose, is the only treatment that reduced the NO_3_^−^ concentration in lettuce leaves with no differences between the rest of the treatments (control and 5 mL L^−1^ Green Leaves) (Figure 4). These results are very interesting from a nutritional point of view because they indicate that the use of Green Leaves at a 3 mL L^−1^ dose would mean an improvement in the nutritional quality of these plants intended for human consumption. It is important to analyze the effect of biostimulant application on NO_3_^−^ accumulation in lettuce, as various results have been found in other studies. Thus, in some experiments, the application of the biostimulant leads to greater NO_3_^−^ accumulation, whereas in others, it reduces it, depending on the type of biostimulant and the conditions of the experiment [21,23].

### 2.6. Concentration and Efficiency of the Use of Mineral Nutrients

**Figure 5:**
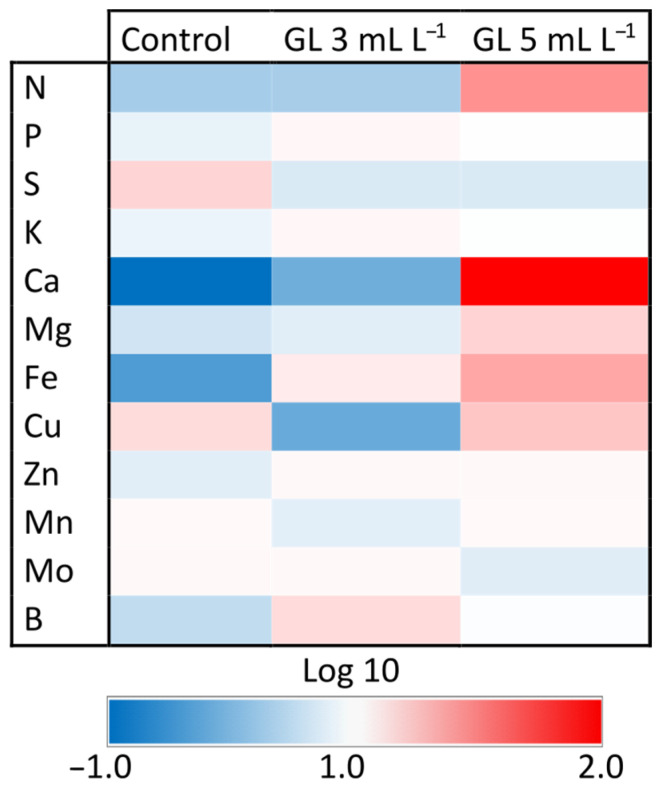
Heat map showing the effect of Green Leaves treatments on the leaf concentration of macronutrients and micronutrients in lettuce plants. Color scale refers to the logarithmic transformation (log10) of measured values (higher values are shown in red, lower values in blue, and intermediate values in white). For the interpretation of the color code, refer to Table S1.

Generally, the mineral content measured in this study falls within the expected ranges, with no significant excesses or deficiencies that would impede the normal growth of plants [52]. Regarding macronutrients, the application of the Green Leaves product only significantly affected the foliar concentrations of N and Ca, with the maximum concentrations occurring with the application of the 5 mL L^−1^ dose (Figure 5; Table S1). These results are possibly due to the exogenous application of organic N compounds and the element Ca that is produced when the highest dose of Green Leaves is used. These N and Ca increments could contribute to the positive response of growth stimulation in plants because N plays a pivotal role in the biochemistry of numerous compounds, whereas Ca is critical for maintaining cell wall and membrane integrity and function. Furthermore, Ca acts as a secondary messenger in hormonal and environmental response mechanisms. The nutritional and physiological importance of N and Ca is fundamental for achieving maximum yields in a wide variety of crops [53].

Considering micronutrients, the application of Green Leaves at both doses only significantly affects the foliar Fe concentration, with both treatments presenting concentrations higher than those obtained in control plants (Figure 5, Table S2). As occurred in the case of N and Ca, this increment in the foliar concentration of Fe may be due to the exogenous application of this element with the Green Leaves product. The increase in Fe in lettuce leaves due to the application of Green Leaves could contribute to improving the physiological state of the plants because Fe plays a pivotal role as a component of the catalytic centers in various hemoprotein redox enzymes, including cytochromes, peroxidases, and catalases. It is also vital for photosynthesis and N assimilation [54].

Another aspect to consider is efficiency in the use of these mineral nutrients, in order for good production to be obtained without excessive application of fertilizers that could cause environmental pollution. Other studies proved that the application of biostimulants can increase nutrient use efficiencies, reducing their contribution without compromising production [55,56]. The parameters proposed by Dobermann [57] were used in this study to define nutrient use efficiency (NUE) in plants. The European Union defined these parameters in the biostimulant regulations listed in FprCEN/TS 17700-2 to certify these products as improving this nutritional characteristic.

**Figure 6:**
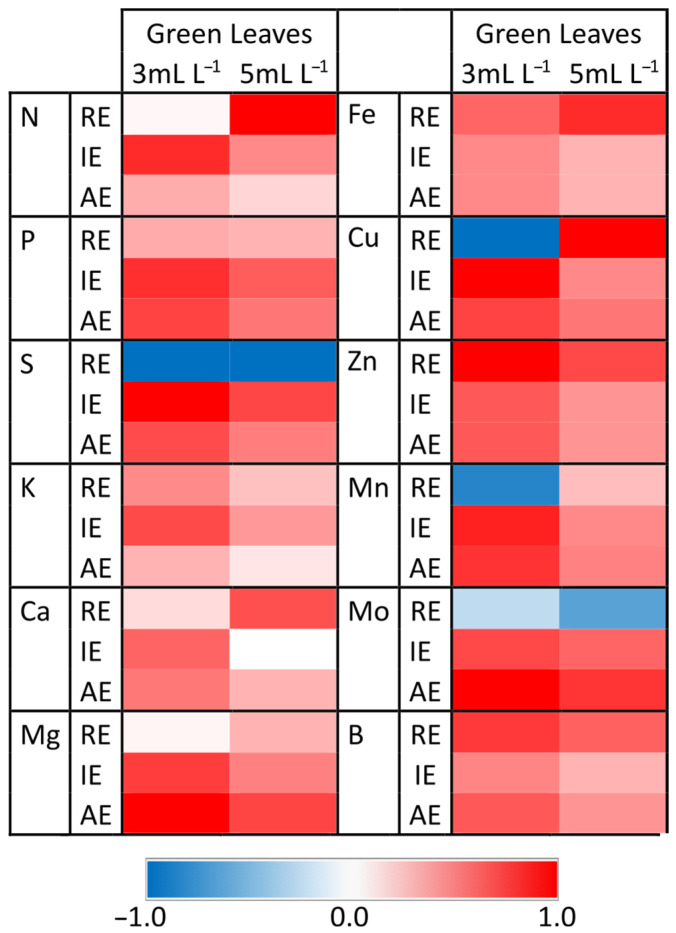
Heat map showing the effect of Green Leaves treatments on the apparent recovery efficiency of the applied nutrient (RE), internal utilization efficiency of the nutrients (IE), and agronomic efficiency of the nutrients applied (AE) in lettuce plants. Color scale refers to the normalized data of measured values (higher values compared to control plants are shown in red, lower values compared to control plants are shown in blue, and similar values to control plants are show in white). For the interpretation of the color code, refer to Tables S3 and S4.

Considering the recovery efficiency of the applied nutrient (RE) parameter, which indicates the absorption efficiency of the different nutrients contained in a fertilizer, we generally found that the application of Green Leaves produced an increase in the RE of most nutrients, except for the elements S, Mo, and Mn, as well as Cu, only for 3 mL L^−1^ dose. Indeed, the positive effect of Green Leaves on RE depends on the applied dose of the product. Thus, the use of the 3 mL L^−1^ dose increased the absorption of nutrients K, Zn, and B, whereas the application of the 5 mL L^−1^ dose had a greater effect on N, Ca, Mg, Fe, Cu, and Mn (Figure 6; Tables S3 and S4). Regarding the IE and AE parameters, the application of Green Leaves at both doses produced positive values for all nutrients compared with the control plants, which indicates that the application of this product benefits the utilization of all essential nutrients for the generation of biomass in lettuce plants. The maximum values in most nutrients were obtained when Green Leaves was applied at the 3 mL L^−1^ dose (Figure 6; Tables S3 and S4). These results are very interesting because the application of the Green Leaves product improved the RE of most nutrients, especially N, which could allow for the cultivation of crops in areas with a deficiency of these nutrients, in addition to allowing for a decrease in the use of fertilizer without reducing crop yields. This reduction, in turn, could reduce costs and environmental impact. Therefore, the Green Leaves product could be a useful tool to reduce the use of fertilizers, especially the more expensive nitrogenous ones with greater environmental impact, which is in line with the European Union’s plants to be established in the near future [58].

## 3. Materials and Methods

### 3.1. Plant Material and Growing Conditions

Lettuce plants (*Lactuca sativa* cv. Maravilla de Verano) were used in the experiment as plant material. The seeds were germinated and cultivated for 45 days in trays divided into cells (3 cm × 3 cm × 10 cm) at Saliplant SL (Carchuna, Granada, Spain). Subsequently, the seedlings were relocated to a controlled environment chamber. The plants grew under specific conditions: a relative humidity of 60–80%, a temperature cycle of 25 °C during the day and 15 °C at night, and a light regimen of 16h of light followed by 8h of darkness. The light intensity (350 µmol m^−2^s^−1^) was provided by fluorescent tubes and was measured using an SB quantum 190 sensor (LI—COR Inc., Lincoln, NE, USA).

Each plant was individually transplanted into separate pots (13 × 10 × 12.5 cm) with a total volume of 2 l. The growing substrate used was a combination of perlite and vermiculite. The plants were watered with a Hogland-type nutrient solution, slightly modified to suit lettuce needs. This solution contained 4 mM KNO_3_, 2 mM Ca(NO_3_)_2_, 2 mM MgSO_4_, 1 mM KH_2_PO_4_, 1 mM NaH_2_PO_4_, 2 µM MnCl_2_, 125 µM Fe-EDDHA, 50 µM H_3_BO_3_, 1 µM ZnSO_4_, 0.25 µM CuSO_4_, and 0.1 µM Na_2_MoO_4_. The solution was adjusted to a pH of 5.8, and each plant received approximately 50 mL daily, ensuring that the volume of drainage did not exceed 10% of the total.

### 3.2. Treatments Description and Experimental Design

The product used in this experiment was Green Leaves, generated by the company Atlántica Agrícola SL, which, in addition to being formed by an extract of *Macrocystis* algae (brown algae), contains a mixture of AAs, such as glutamate and glycine, corn steep liquor extract, the macronutrient Ca, and the bioactive compound glycine betaine.

In this experiment, the effect of foliar application of the Green Leaves product at two different doses (3 mL L^−1^ and 5 mL L^−1^) was analyzed. A group of control plants was established to which the Green Leaves product was not applied. Treatment administration commenced 7 days after transplantation into the growth chamber. Following Atlántica Agrícola SL’s R&D team guidelines, the test product was applied twice, maintaining a seven-day interval between the two applications. This study used a completely randomized block design, featuring three replicates for each treatment. These replications included trays holding eight plants each, which were individually potted and randomly allocated within the growth chamber for treatment application.

### 3.3. Vegetable Sampling

Shoot samples were collected 7 days after the final treatment application. Immediately upon collection, all plants from each treatment group were prepared for further analysis. This involved rinsing the plant material with distilled water and then drying it on filter paper and weighing it to measure the fresh weight (FW). Subsequently, one half of the plant material was frozen at −40 °C for the assessment of photosynthetic pigment concentration, concentrations of AAs, proteins, soluble sugars, FRAP and TEAC antioxidant tests, concentration of flavonoids, anthocyanins, total phenols, ascorbate, and nitrates. The remaining half of the plant material was desiccated using a freeze-drying process to determine the dry weight (DW) and analyze the concentration of mineral nutrients.

### 3.4. Plant Material Analysis

#### 3.4.1. Leaf Area

Leaf area per plant was analyzed using a LI-COR optical reader, model LI-3000A.

#### 3.4.2. Chlorophyll (Chl) a Fluorescence

Before conducting measurements, the leaves underwent a 30 min acclimatization period in complete darkness. This was achieved by attaching a specialized leaf clip to each leaf. The dynamics of chlorophyll (Chl) *a* fluorescence were evaluated using a Handy PEA Chl Fluorimeter (Hansatech Ltd., King’s Lynn, Norfolk, UK). This process involved the initiation of OJIP fluorescence phases through the exposure of the leaves to red light at 650 nm and an intensity of 3000 µmol photons m^−2^s^−1^. Analysis of the OJIP fluorescence phases was performed employing the JIP test methodology, as outlined by Strasser et al. [32]. Measurements targeted fully mature leaves located at the median section of the plant, focusing on evaluating energy transfer and photosynthetic efficiency via parameters derived from the JIP test: initial fluorescence (Fo), maximum fluorescence (Fm), variable fluorescence (Fv = Fm − Fo), maximum quantum product of primary photochemistry (Fv/Fm), performance index (PI_ABS_), proportion of active reaction centers (RC) (RC/ABS), and the efficiency with which a trapped exciton moves an electron further than quinone A into the electron transport chain (ψ_o_) [32].

#### 3.4.3. Gas Exchange Parameters

Data collection was performed using a LICOR 6800 Portable Photosynthesis System Infrared Gas Analyzer (IRGA, LICOR Inc., Nebraska, USA). Leaves from the middle section of plants were analyzed in measurement chambers provided by the system, set to conditions that mirrored this ideal for growth. The device was preheated for 30 min and calibrated before the measurements began. The experimental setup included a photosynthetically active radiation (PAR) of 500 μmol m^2^ s^−1^, a CO_2_ concentration of 400 μmol mol^−1^, a leaf temperature maintained at 30 °C, and a relative humidity of 60%. The system simultaneously captured the net photosynthetic rate (*A*), transpiration rate (*E*), and stomatal resistance (r), storing these data for subsequent analysis using the “Photosyn Assistant” software (Version 3). The instantaneous water use efficiency (WUE) was determined by the ratio of *A* to *E*.

#### 3.4.4. Concentration of Photosynthetic Pigments

The Wellburn [59] method with slight modifications was used. Pigments were extracted with methanol, centrifuged for 5 min at 5000× *g*, and the absorbance was ultimately measured at 666, 653, and 470 nm. The following formulas were made based on the following equations:

- Chl *a* = 15.65 × A666 nm − 7.34 × A653 nm;
- Chl *b* = 27.05 × A653 nm − 11.21 × A666 nm;
- Carotenoids = (1000 × A470 nm − 2.86 × Chl *a* − 129.2 × Chl *b*)/221.

#### 3.4.5. Determination of Amino Acids, Proteins, and Soluble Sugars

For the analysis of AAs and soluble proteins, the methodology described in Navarro-León et al. [60] was followed. Soluble AAs analysis was based on the reaction with ninhydrin and reading absorbance at 570 nm, and soluble protein analysis was based on the reaction with Coomassie blue and absorbance registered at 595 nm. For the concentration of soluble sugars, the method of Irigoyen et al. [61] was followed based on the reaction with anthrone and reading the absorbance at 650 nm.

#### 3.4.6. Antioxidant Capacity: FRAP and TEAC Tests

The FRAP test was based on the reaction with 2,4,6-tripyryldyl-2-triazine (TPTZ) and 20 mM FeCl_3_ and a final recording of absorbance at 593 nm [62]. The TEAC test was performed according to Cai et al. [63] based on the reaction with 2,2’-azinobis-(3-ethylbenzothiazoline-6-sulfonic acid) (ABTS) and the final recording of absorbance at 734 nm.

#### 3.4.7. Determination of Total Phenol, Flavonoid, and Anthocyanin Concentrations

The quantification of total phenols in the plant samples was performed using a method adapted from Rivero et al. [64], involving the extraction of plant material with a solution of methanol, chloroform, and NaCl. The quantification was completed using the Folin-–Ciocalteu reaction, with absorbance readings taken at 725 nm to determine phenolic content. For total flavonoid content, the procedure outlined by Kim et al. [65], with certain modifications, was employed. This involved the same extraction method as used for phenols, with the resulting extract treated with NaNO_2_ and AlCl_3_, and absorbance measured at 415 nm to assess flavonoid concentration.

The measurement of anthocyanin levels was conducted through the differential pH method as described by Giusti et al. [66]. This process involved extracting the plant material with acidified methanol, followed by centrifugation. The supernatant was then mixed with potassium chloride, and its absorbance was determined using a spectrophotometer at 460 nm. Secondly, the supernatant was mixed with sodium acetate and measured at an absorbance of 710 nm. To obtain the concentration of anthocyanins in the different varieties of cauliflower, the following formula was applied:

[((A460 − A710) × 449.2 × 0.2 × 1000)/26,900]

#### 3.4.8. Determination of Ascorbate Concentration

For ascorbate quantification, the method of Law et al. [67] was followed. This method is based on the reduction of Fe^3+^ to Fe^2+^ by ascorbate in an acidic solution. The leaf sample was subjected to extraction with metaphosphoric acid and subsequently centrifuged. The resulting extract was then reacted with a mixture of N-ethylmaleimide, trifluoroacetic acid, orthophosphoric acid, bipyridyl, and iron chloride. Finally, the absorbance of the resulting colored compound was measured at 525 nm by spectrophotometry.

#### 3.4.9. Determination of Nitrates

Quantification of soluble NO_3_^−^ levels was achieved through an aqueous extraction process, adhering to the protocol established by Cataldo et al. [68]. The aqueous extract was mixed with 10% salicylic acid diluted in H_2_SO_4_ and with 2N NaOH. Subsequently, the mixture was stirred, resulting in a color change. Finally, the absorbance of the samples was measured at 410 nm by spectrophotometry.

#### 3.4.10. Mineral Nutrient Concentrations

The analysis of nutrient concentrations, specifically P, K, Ca, Mg, S, Fe, Cu, Mn, Zn, Mo, and B in leaf samples, was conducted using inductively Coupled Plasma Optical Emission Spectroscopy (ICP-OES). Following the Wolf [69] methodology, the samples underwent a mineralization process, where 0.2 g of dried leaf and root material was digested in a mixture of 30% nitric acid (HNO_3_) and hydrogen peroxide (H_2_O_2_) at 300 °C to prepare the samples for ionic element analysis. For total nitrogen (N) content determination, a similar quantity of dried samples was finely ground and mineralized using 98% sulfuric acid (H_2_SO_4_) and 30% H_2_O_2_ at 300 °C. The resultant solution was analyzed for N concentration using colorimetric techniques based on the Berthelot reaction, as outlined by Krom [70].

#### 3.4.11. Nutrient Use Efficiency Parameters

To calculate the efficiency in the use of nutrients (N), the formulas defined by Dobermann [57] were used, which are the basis of the European regulation of Biostimulants FprCEN/TS 17700-2, as indicated here:

RE = Apparent recovery efficiency of applied N: RE = ([N]_Treated plant_ − [N]_Control_ plant)/[N]_Applied_) = mg N;

IE = Internal utilization efficiency of the nutrient (N): IE = Biomass/[N_organ or complete plant_] = g^2^ dry matter/mg N leaf;

AE = Agronomic efficiency of N applied: AE = (Biomass_Treatment_–Biomass_Control_)/[N]_Applied_ = g dry matter/mg N applied.

### 3.5. Statistical Analysis

The collected data underwent statistical examination using variance analysis and one way ANOVA, all within a 95% confidence interval. To discern any significant differences among the treatment averages, Fisher’s Least Significant Difference (LSD) test was employed at a 95% confidence level. Significance was denoted as follows: * *p* < 0.05; ** *p* < 0.01; *** *p* < 0.001; NS not significant. Statgraphics Centurion 16.1.03 software was used to perform statistical analysis.

## 4. Conclusions

The application of Green Leaves, in two tested concentrations, significantly enhanced the growth of lettuce plants. It boosted the photosynthetic rate, accumulation of soluble sugars and proteins, and the agronomic efficiency of all essential nutrients. Notably, the 3 mL L^−1^ concentration enriched the nutritional quality of lettuce by increasing the concentration of antioxidant compounds, enhancing antioxidant capacity, and reducing nitrate (NO_3_^−^) accumulation. On the other hand, the 5 mL L^−1^ concentration proved to be more effective in enhancing the uptake of most nutrients, particularly N, which could decrease the necessity for fertilizers, thereby not only preserving crop yields but also reducing costs and environmental impacts. Overall, the Green Leaves product has been identified as a beneficial tool for achieving higher yields and improved quality, while also diminishing the need for fertilizer applications. Future studies should explore the specific physiological mechanisms activated by the biostimulant in greater detail, and also assess its effectiveness in improving plant tolerance to abiotic stress.

## Figures and Tables

**Table 1:** Effect of Green Leaves treatments on fresh and dry weights and the leaf area of lettuce plants.

Treatments	Fresh Weight (g)	Dry Weight (g)	Leaf Area (cm^2^)
Control	39.57 ± 1.46 b	1.68 ± 0.13 b	651.61 ± 18.62 b
Green Leaves 3 mL L^−1^	48.50 ± 2.14 a (+23%)	2.46 ± 0.08 a (+46%)	698.43 ± 14.73 a
Green Leaves 5 mL L^−1^	49.74 ± 0.79 a (+26%)	2.29 ± 0.07 a (+36%)	684.26 ± 29.07 a
*p*-value	**	**	**

% data indicate increase (+) or decrease (−) with respect to the values of control plants. All data values represent means ± standard error. The level of significance was represented as ** (*p* < 0.01). Values with different letters indicate significant differences.

**Table 2:** Effect of Green Leaves treatments on chlorophyll *a* fluorescence parameters in lettuce plants.

Treatments	Fv/Fm	RC/ABS	PI_ABS_	Ψ_o_
Control	0.866 ± 0.002	0.330 ± 0.004	2.133 ± 0.053	0.501 ± 0.004
Green Leaves 3 mL L^−1^	0.867 ± 0.002	0.322 ± 0.007	2.201 ± 0.067	0.511 ± 0.004
Green Leaves 5 mL L^−1^	0.868 ± 0.002	0.321 ± 0.002	2.213 ± 0.102	0.511 ± 0.008
*p*-value	NS	NS	NS	NS

Variable fluorescence/maximum fluorescence ratio (Fv/Fm), proportion of active reaction centers (RC/ABS), performance index (PI_ABS_), and efficiency with which a trapped exciton moves an electron further than quinone A into the electron transport chain (Ψo). Values are means ± standard deviation. The levels of significance were represented as non-significant (NS) (*p* > 0.05).

**Table 3:** Effect of treatments on the concentration of photosynthetic pigments in lettuce plants.

Treatments	Chl *a* (mg g^−1^ FW)	Chl *b* (mg g^−1^ FW)	Carotenoids (mg g^−−1^ FW)
Control	216.51 ± 6.25	118.13 ± 1.68	26.20 ± 1.54
Green Leaves 3 mL L^−1^	198.83 ± 2.33	115.54 ± 2.34	23.72 ± 0.58
Green Leaves 5 mL L^−1^	211.49 ± 1.28	120.36 ± 1.67	23.92 ± 0.70
*p*-value	NS	NS	NS

Values are means ± standard deviation. The levels of significance were represented as non-significant (NS) (*p* > 0.05).

**Table 4:** Effect of treatments on the parameters of photosynthetic efficiency and gas exchange in lettuce plants.

Treatments	*A* (µmol m^−2^ s^−1^)	*E* (mmol m^−2^ s^−1^)	C_i_ (µmol mol^−1^)	r (s cm^−1^)	WUE
Control	7.13 ± 0.20 b	1.90 ± 0.07 b	261.69 ± 3.61 b	8.98 ± 0.53 a	3.77 ± 0.08
Green Leaves 3 mL L^−1^	8.94 ± 0.16 a	2.69 ± 0.09 a	280.66 ± 1.06 a	7.14 ± 0.40 c	3.34 ± 0.11
Green Leaves 5 mL L^−1^	9.08 ± 0.14 a	2.51 ± 0.09 a	275.46 ± 3.67 a	7.73 ± 0.29 b	3.64 ± 0.12
*p*-value	**	**	**	***	NS

Net photosynthetic rate (*A*), intercellular CO_2_ (C_i_) transpiration rate (*E*), stomatal resistance (r), water use efficiency (WUE). Values are means ± standard deviation. The level of significance was represented as non-significant (NS) (*p* > 0.05), ** (*p* < 0.01), and *** (*p* < 0.001). Values with different letters indicate significant differences.

**Table 5:** Effect of treatments on some nutritional quality parameters in lettuce plants.

Treatments	Total Phenols (mg g^−1^ FW)	Flavonoids (mg g^−1^ FW)	Anthocyanins (µg g^−1^ FW)	Ascorbate (µg g^−1^ FW)
Control	0.33 ± 0.01 c	0.24 ± 0.04b	23.45 ± 1.52 c	33.80 ± 4.07 c
Green Leaves 3 mL L^−1^	0.47 ± 0.01 a	0.42 ± 0.01 a	72.96 ± 3.42 a	55.64 ± 4.41 a
Green Leaves 5 mL L^−1^	0.40 ± 0.01 b	0.39 ± 0.01 a	64.71 ± 1.45 b	45.02 ± 6.78 b
*p*-value	**	***	***	**

Values are means ± standard deviation. The level of significance is represented as ** (*p* < 0.01) and *** (*p* < 0.001). Values with different letters indicate significant differences.

## Data Availability

The datasets generated and analyzed during this current study are available from the corresponding author upon reasonable request.

## Supplementary Materials

The following supporting information can be downloaded at https://www.mdpi.com/article/10.3390/plants13070917/s1, Table S1: Effect of treatments on macronutrient concentration in lettuce plants; Table S2: Effect of treatments on micronutrient concentration in lettuce plants; Table S3: Effect of treatments on macronutrient efficiency parameters in lettuce plants; Table S4: Effect of treatments on micronutrient efficiency parameters in lettuce plants.
