# Supplementary material for: An Evaluation of the Effectivity of the Green Leaves Biostimulant on Lettuce Growth, Nutritional Quality, and Mineral Element Efficiencies under Optimal Growth Conditions
Source: Plants (Basel). 2024 Mar 22;13(7):917. doi: 10.3390/plants13070917 (PMC11013046; doi:10.3390/plants13070917)
Supplement: Supplementary file 1 [file plants-13-00917-s001.zip › plants-2907183-supplementary.pdf]

Table S1. Effect of treatments on macronutrient concentration in lettuce plants.

**Treatments**	N	P	S	K	Ca	Mg
Control	65.94±1.18b	9.72±0.26	2.92±0.06	37.21±0.20	11.54±0.31c	7.39±0.25
Green Leaves 3 mL L^{-1}	66.00±0.44b	9.91±0.07	2.76±0.02	37.86±0.04	12.36±0.35b	7.46±0.06
Green Leaves 5 mL L^{-1}	75.75±3.08a	9.85±0.03	2.76±0.01	37.57±0.01	16.29±0.38a	7.87±0.03
p–value	*	NS	NS	NS	***	NS

Values are expressed as mg g^{-1} DW. Values are means ± standard deviation. The level of significance was represented as non–significant (NS) ($p > 0.05$) and *** ($p < 0.001$). Values with different letters indicate significant differences.

Table S2. Effect of treatments on micronutrient concentration in lettuce plants.

**Treatments**	**Fe**	**Cu**	**Zn**	**Mn**	**Mo**	**B**
Control	79.03±21.02c	14.71±0.33	44.45±1.21	150.45±4.95	0.44±0.01	30.49±1.55
Green Leaves 3 mL L^{-1}	89.11±2.23b	13.11±0.14	45.09±1.35	147.06±1.45	0.44±0.01	32.28±0.45
Green Leaves 5 mL L^{-1}	94.42±3.05a	14.98±0.03	45.67±1.92	150.68±2.69	0.43±0.01	31.03±0.27
p–value	***	NS	NS	NS	NS	NS

Values are expressed as μg g^{-1} DW. Values are means ± standard deviation. The level of significance was represented as non–significant (NS) ($p > 0.05$) and *** ($p < 0.001$). Values with different letters indicate significant differences.

Table S3. Effect of treatments on macronutrient efficiency parameters in lettuce plants.

		**N**	
	**RE**	**IE**	**AE**
Control	–	0.025±0.001c	–
Green Leaves 3 mL L^{-1}	0.06±0.43b	0.037±0.001a	0.77±0.05a
Green Leaves 5 mL L^{-1}	9.71±3.06a	0.031±0.002b	0.60±0.04b
p–value	***	***	**
		**P**	
	**RE**	**IE**	**AE**
Control	–	0.17±0.01b	–
Green Leaves 3 mL L^{-1}	0.33±0.13	0.25±0.01a	1.40±0.09a
Green Leaves 5 mL L^{-1}	0.24±0.06	0.23±0.01a	1.09±0.08b
p–value	NS	**	***
		**S**	
	**RE**	**IE**	**AE**
Control	–	0.56±0.04c	–
Green Leaves 3 mL L^{-1}	−0.29±0.04	0.89±0.02a	1.35±0.08a
Green Leaves 5 mL L^{-1}	−0.29±0.02	0.83±0.02b	1.05±0.08b
p–value	NS	***	**
		**K**	
	**RE**	**IE**	**AE**
Control	–	0.05±0.00b	–
Green Leaves 3 mL L^{-1}	0.46±0.03a	0.07±0.00a	0.56±0.03a
Green Leaves 5 mL L^{-1}	0.25±0.01b	0.06±0.00b	0.43±0.03b
p–value	**	*	***
		**Ca**	
	**RE**	**IE**	**AE**
Control	–	0.15±0.01b	–
Green Leaves 3 mL L^{-1}	1.14±0.49b	0.20±0.01a	1.08±0.07a
Green Leaves 5 mL L^{-1}	6.59±0.53a	0.14±0.00b	0.84±0.06b
p–value	***	*	**
		**Mg**	
	**RE**	**IE**	**AE**
Control	–	0.23±0.02c	–
Green Leaves 3 mL L^{-1}	0.14±0.14b	0.33±0.01a	1.79±0.11a
Green Leaves 5 mL L^{-1}	1.07±0.08a	0.29±0.01b	1.39±0.10b
p–value	**	***	**

Apparent recovery efficiency of applied nutrient (RE), internal utilization efficiency of the nutrient (IE), and agronomic efficiency of nutrient applied (AE). Values are means ± standard deviation. The level of significance was represented as non–significant (NS) ($p > 0.05$), * ($p < 0.05$), ** ($p < 0.01$), and *** ($p < 0.001$). Values with different letters indicate significant differences.

Table S4. Effect of treatments on micronutrient efficiency parameters in lettuce plants.

		Fe	
	**RE**	**IE**	**AE**
Control	–	0.021±0.001c	–
Green Leaves 3 mL L^{-1}	165.99±32.36b	0.028±0.001a	12.41±0.76a
Green Leaves 5 mL L^{-1}	228.55±45.07a	0.025±0.001b	9.63±0.70b
p–value	***	***	***
		**Cu**	
	**RE**	**IE**	**AE**
Control	–	0.11±0.01c	–
Green Leaves 3 mL L^{-1}	−11404±987b	0.19±0.00a	5583±344a
Green Leaves 5 mL L^{-1}	1989±182a	0.15±0.00b	4333±314b
p–value	***	***	***
		**Zn**	
	**RE**	**IE**	**AE**
Control	–	0.038±0.002c	–
Green Leaves 3 mL L^{-1}	1984±236a	0.055±0.001a	1325±82a
Green Leaves 5 mL L^{-1}	1121±196b	0.051±0.001b	1028±74b
p–value	***	***	***
		**Mn**	
	**RE**	**IE**	**AE**
Control	–	0.011±0.001c	–
Green Leaves 3 mL L^{-1}	−2615±1280b	0.017±0.000a	789±49a
Green Leaves 5 mL L^{-1}	186.17±1678a	0.015±0.000b	613±44b
p–value	**	**	***
		**Mo**	
	**RE**	**IE**	**AE**
Control	–	3.83±0.17b	–
Green Leaves 3 mL L^{-1}	−42.34±87.58	5.65±0.14a	9089±559a
Green Leaves 5 mL L^{-1}	−122.15±82.74	5.33±0.14a	7054±511b
p–value	NS	*	***
		**B**	
	**RE**	**IE**	**AE**
Control	–	0.057±0.004c	–
Green Leaves 3 mL L^{-1}	373.73±81.99a	0.078±0.001a	159.52±9.82a
Green Leaves 5 mL L^{-1}	263.35±43.28b	0.074±0.001b	123.81±8.97b
p–value	**	**	***

Apparent recovery efficiency of applied nutrient (RE), internal utilization efficiency of the nutrient (IE), and agronomic efficiency of nutrient applied (AE). Values are means ± standard deviation. The level of significance was represented as non−significant (NS) ($p > 0.05$), * ($p < 0.05$), ** ($p < 0.01$), and *** ($p < 0.001$). Values with different letters indicate significant differences.
